# Cancer risk assessment of ethyl carbamate in alcoholic beverages from Brazil with special consideration to the spirits cachaça and tiquira

**DOI:** 10.1186/1471-2407-10-266

**Published:** 2010-06-08

**Authors:** Dirk W Lachenmeier, Maria CP Lima, Ian CC Nóbrega, José AP Pereira, Florence Kerr-Corrêa, Fotis Kanteres, Jürgen Rehm

**Affiliations:** 1 Chemisches und Veterinäruntersuchungsamt (CVUA) Karlsruhe, Weissenburger Strasse 3, D-76187 Karlsruhe, Germany; 2 Departamento de Neurologia, Psicologia e Psiquiatria, Botucatu Medical School UNESP, São Paulo State University, CP 540 - Distrito Rubiao Junior, 18618-970 Botucatu, São Paulo, Brazil; 3 Universidade Federal Rural de Pernambuco, Programa de Pós-graduação em Ciência e Tecnologia de Alimentos, CEP 52171-900, Recife, PE, Brazil; 4 Centre for Addiction and Mental Health (CAMH), 33 Russell Street, Toronto, ON, M5 S 2S1, Canada; 5 Faculty of Health, Medicine and Life Sciences, Maastricht University, P.O. Box 616, 6200 MD Maastricht, The Netherlands; 6 Dalla Lana School of Public Health, University of Toronto, 55 College Street, Toronto, ON, M5T 3M7, Canada; 7 Epidemiological Research Unit, Institute for Clinical Psychology and Psychotherapy, TU Dresden, Chemnitzer Strasse 46, 01187 Dresden, Germany

## Abstract

**Background:**

Ethyl carbamate (EC) is a multi-site carcinogen in experimental animals and probably carcinogenic to humans (IARC group 2A). Traces of EC below health-relevant ranges naturally occur in several fermented foods and beverages, while higher concentrations above 1 mg/l are regularly detected in only certain spirits derived from cyanogenic plants. In Brazil this concerns the sugarcane spirit cachaça and the manioc (cassava) spirit tiquira, which both regularly exceed the national EC limit of 0.15 mg/l. This study aims to estimate human exposure in Brazil and provide a quantitative risk assessment.

**Methods:**

The human dietary intake of EC via alcoholic beverages was estimated based on WHO alcohol consumption data in combination with own surveys and literature data. This data comprises the EC contents of the different beverage groups cachaça, tiquira, other spirits, beer, wine, and unrecorded alcohol (as defined by the WHO; including alcohol which is not captured in routine government statistics nor taxed). The risk assessment was conducted using the margin of exposure (MOE) approach with benchmark doses obtained from dose-response modelling of animal experiments. Lifetime cancer risk was calculated using the T25 dose descriptor.

**Results:**

Considering differences between pot-still and column-still cachaça, its average EC content would be 0.38 mg/l. Tiquira contained a considerably higher average EC content of 2.34 mg/l. The whole population exposure from all alcoholic beverages was calculated to be around 100 to 200 ng/kg bw/day, with cachaça and unrecorded alcohol as the major contributing factors. The MOE was calculated to range between 400 and 2,466, with the lifetime cancer risk at approximately 3 cases in 10,000. An even higher risk may exist for binge-drinkers of cachaça and tiquira with MOEs of up to 80 and 15, respectively.

**Conclusions:**

According to our risk assessment, EC poses a significant cancer risk for the alcohol-drinking population in Brazil, in addition to that of alcohol alone. Model calculations show that the implementation of the 0.15 mg/l limit for cachaça would be beneficial, including an increase of the MOE by a factor between 3 to 6. The implementation of policy measures for tiquira and unrecorded alcohol also appears to be advisable.

## Background

According to epidemiological findings, the consumption of alcoholic beverages is causally related to the occurrence of malignant tumours of the oral cavity, pharynx, larynx, oesophagus, liver, colorectum, and female breast; this includes a classification as "carcinogenic to humans" (group 1) by the IARC [1]. Because the carcinogenicity was generally noted with different types of alcoholic beverage, and in view of the carcinogenicity of ethanol in animals, the IARC also classified ethanol in alcoholic beverages as "carcinogenic to humans" (group 1) [1]. The major mechanism appears to be the metabolism of ethanol to acetaldehyde, which was proven by genetic-epidemiological evidence as a risk-factor for alcohol-related oesophageal and head and neck cancers, and acetaldehyde associated with alcohol consumption was also recently upgraded to group 1 by the IARC [2].

While there is consensus that ethanol along with acetaldehyde are the major carcinogenic factors in alcoholic beverages, other constituents and contaminants may additionally contribute to the carcinogenicity, especially in unrecorded alcohol that is not quality controlled [3]. From the range of possible carcinogenic substances in alcoholic beverages (e.g. lead, nitrate, certain pesticides and mycotoxins), EC (ethyl carbamate, urethane, C_2_H_5_OCONH_2_, CAS # 51-79-6) is the most likely candidate for causing additional carcinogenicity, and has been judged as a probable health risk for regular drinkers of certain types of alcoholic beverages (see below) [4,5].

EC is a recognized genotoxic carcinogen, with widespread occurrence in fermented foods and beverages [6-11]. In rodents, EC has been demonstrated to cause dose-dependent increases in liver, lung, and harderian gland adenoma or carcinoma, and hemangiosarcoma of the liver and heart (both sexes), mammary gland and ovarian tumours (females), and squamous cell papilloma as well as carcinoma of the skin and forestomach (males). The increase in hepatocellular tumours was found to occur in a relatively linear manner and was attributed to the formation of 1, *N*^6^-ethenodeoxyadenosine in hepatic DNA coupled with an increase in cell replication. Lung alveolar/bronchiolar and harderian gland adenoma or carcinoma also increased in a relatively linear manner, suggesting a genotoxic mechanism for tumour induction [12]. In 2007, the IARC upgraded its classification of EC to group 2A (probably carcinogenic to humans) [13]. This reflects mounting evidence regarding the metabolic pathways of the activation of EC, wherein the formation of proximate DNA-reactive carcinogens, hypothesized to play a major role in EC-induced carcinogenesis in rodent cells, is also likely to occur in humans due to significant similarities with rodents [13]. Limited evidence from the human administration of EC has shown that it can in fact cause liver disease, specifically hepatic angiosarcoma [14].

While the concentration of EC in foods and most beverage groups is very low and not seen as a public health risk [4,5], concerns about the presence of this substance in alcoholic beverages began in 1985, when comparably high levels were detected by Canadian authorities in imported alcohol products [15]. Canada proceeded to establish an upper limit of 0.15 mg/l for EC in distilled spirits [4]. The rationale for the Canadian limit was based on a VSD of 0.3 μg/kg, a level that, in combination with a daily intake of 125 g distilled spirits, would not result in an increased incidence of cancer greater than one in a million [15]. The VSD was extrapolated from the daily dose required to produce tumours in 50% of the exposed animals over a standard lifetime and adjusted for background incidence. The same 0.15 mg/l limit is currently being established in Brazil for cachaça, to be enacted in June 2010 [16]. In this context, our team recently detected that 70% of the analyzed products from Paraíba State, Brazil, exceeded this limit [17]. The relatively high incidence for EC contamination was confirmed in a second survey by our group, in which 55% of all samples were above 0.15 mg/l [18].

International risk assessments of EC in alcoholic beverages have found that exposure levels may exceed acceptable thresholds. For example, the JECFA [5] estimated the MOE, the risk assessment approach also preferred by the EFSA, as being 3,800 in certain alcoholic beverages. The MOE approach has been advised by the JECFA and EFSA as the best approach for assessing substances that are both genotoxic and carcinogenic. The MOE is defined as the ratio between the point on the dose response curve which characterizes adverse effects in animal studies, and the estimated human intake of the same compound. Clearly, the lower the MOE, the larger the risk for humans. A threshold of 10,000 is often used to define public health risks. In this framework, 3,800 is a value of concern, necessitating mitigative measures, namely lowering the concentration of the substance. The EFSA also recently confirmed the JECFA evaluation, noting that the MOE for high consumers of fruit spirits was less than 600, indicating an even greater public health concern [4]. The JECFA evaluation was based on data from the USA, the UK and Japan, whereas the EFSA evaluated data from Europe and North America. Evidence in the international literature (as summarized in Ref. [13]), which is generally restricted to European-style alcoholic beverages, indicates that the EC problem may be limited to certain fruit spirits (mainly stone-fruit spirits). Only recently, the mentioned studies from Brazil pointed to the fact that EC in the sugarcane spirit cachaça may be as problematic as from European fruit spirits, due to the cyanogenic nature of sugarcane materials, with hydrogen cyanide suspected as a major precursor of EC formation in both stone-fruit spirits and cachaça [18]. Another Brazilian spirit described to contain comparably high contamination levels of EC is tiquira, a product derived from manioc/cassava (*Manihot esculenta *Crantz) fermentations [19,20]. Tiquira also contains hydrogen cyanide and other nitrogen compounds discussed as precursors of EC formation [21,22].

Due to the limited presence of cachaça and complete market absence of tiquira in Europe and North America, neither the JECFA nor the EFSA have considered these two types of spirits in their risk assessments. In Brazil, however, where cachaça is the major spirit of consumption (while tiquira consumption is concentrated in the States of Maranhão and Piauí [23]), a quantitative population-based risk assessment is needed. This is even more pertinent, as critical opinions are currently being voiced against the implementation of the EC limit for cachaça in 2010. This article will be the first to provide a quantitative population-based risk assessment using the MOE model established by the JECFA and EFSA. Additionally, we will calculate the lifetime cancer risks for consumers of cachaça, tiquira and beer using the T25 method. Our risk assessment will also be used to evaluate the effectiveness of the 0.15 mg/l limit to protect consumers of alcoholic beverages from additional cancer risks above the risk of ethanol and/or acetaldehyde in alcoholic beverages alone [1].

## Methods

### Literature research

The literature search was conducted by researchers with qualifications in food science, chemistry, toxicology, epidemiology and cancer risk assessment. Data on the occurrence of EC in Brazilian cachaça were obtained by a computer-assisted literature search using the key words 'ethyl carbamate' or 'urethan(e)' and 'cachaça', 'tiquira', 'Brazil' or 'Brazilian'. Benchmark doses and toxicological dose descriptors were obtained by searching with the key words 'ethyl carbamate' or 'urethan(e)' and 'margin of exposure', 'MOE', 'benchmark dose', 'BMD', 'BMDL', 'BMDL10' or 'T25'. Searches in English and Portuguese were carried out in July 2009, in the following databases: PubMed (U.S. National Library of Medicine, Bethesda, MD), Web of Science (Thomson Scientific, Philadelphia, PA), and SciELO - Scientific Electronic Library Online (FAPESP - BIREME, São Paulo SP - Brazil). Efforts were made to include all available studies; this was accomplished by a hand search of the reference lists of all articles for any relevant studies not included in the databases. The references, including abstracts, were imported into Reference Manager V.12 (Thomson Reuters, Carlsbad, CA) and the relevant articles were manually identified and purchased in full text. We did not identify any article, which was available as abstract only or which we were not able to obtain in full text. No unpublished study was identified. If raw data on EC were not present in the article, the corresponding authors were contacted by e-mail with a request for the data.

### Sampling of cachaça in Pernambuco State

Duplicate samples of 33 brands of cachaças (n = 66) produced in Pernambuco State, the second major Brazilian state in terms of production and consumption of cachaça [24], were obtained from retail outlets in Recife, Pernambuco's capital, between February and March 2009. Information on the distillation methods (pot still or column still) was obtained from local inspecting authorities or by visiting distilleries. EC measurements were conducted according to Nóbrega et al. [17].

### Survey of drinking behaviours for individual-based exposure assessment

In order to assess individual exposure scenarios, drinking behaviours and patterns were investigated. The focus was set on lower socioeconomic class for which cachaça, mainly the industrial/column still type, is the typical drink due to its relatively lower price compared to other types of alcoholic beverages [25-28]. For this reason, 17 cachaça selling bars in 5 different low socioeconomic neighbourhoods in Recife were visited in August 2009. This included questioning of patrons regarding typical consumption behaviour and volumetric measurement of common drink sizes. The standard glass size for cachaça was a 190 ml 'copo americano' glass. Customers usually ordered cachaça by single shot or by 'quartinho'. The single shot size varied from 50-80 ml, with the quartinho size approximately 170 ml (an almost full copo americano glass). Prices for the most consumed brands ranged between US$ 0.25-0.50 for a single shot, and US$ 0.75-1.00 for a quartinho shot. Typical consumption patterns included the ingestion of 1-4 shots per drinking occasion (i.e. 50-320 ml) on a weekend basis (i.e. on each Friday, Saturday and Sunday). Daily consumers of cachaça typically consumed 1-2 single shots (i.e. 50-160 ml). Binge drinkers would consume 1-4 quartinhos per drinking occasion (i.e., 170-680 ml). The frequency of such binge-drinking sessions was once or twice a week.

The consumption of cachaça was often followed by that of beer. Beer was commonly served in 600 ml bottles, with typical consumption patterns including 1-3 bottles per drinking occasion (600-1800 ml). Binge drinkers would consume 3-6 bottles per drinking occasion (1800-3600 ml).

### Approach for risk analysis

The risk analysis was conducted according to the harmonised approach of the EFSA for the risk assessment of substances that are genotoxic and carcinogenic [29]. As discussed, the EFSA has developed and recommends an approach known as the MOE. This approach uses the doses of substances that have been observed to cause low but measurable harmful responses (i.e. cancer incidences in this context) in animals as reference point values and, taking into account differences in consumption patterns, compares them with relevant substance specific dietary intake estimates in humans. The BMD, derived from animal cancer data by mathematical modelling within the observed range of experimental data, is recommended as a standardized reference point. To obtain the MOE, the Benchmark Dose Lower Confidence Limit (BMDL) of 10% was taken. The BMDL is an estimate of the lowest dose that is 95% certain to cause no more than a 10% cancer incidence in rodents. In general, BMDLs are used as the statistical lower confidence limits of benchmark doses to derive "safe" exposure levels [30]. MOEs were calculated by dividing the reference point, i.e. the BMDL, by the estimated human intakes.

### Calculation of lifetime cancer risk

While the MOE method is preferred by JECFA and EFSA for risk assessment analysis, the resulting values are dimensionless, with the ratio between rodent carcinogenic dose and human intake not easily interpreted. Thus for a second, more descriptive indicator, we calculated the lifetime cancer risk using the T25 method [31]. The T25 dose descriptor corresponds to the dose representing a 25% incidence of tumours, after correction for spontaneous incidence. The basic difference between the determination of the BMD and the T25 calculation, is that the BMD is accomplished through dose-response modelling that considers all available information on the dose response curve, whereas the T25 is calculated from a single data point [29]. For further details on the T25 calculation see Dybing et al. [32].

### Population-based dietary intake assessment and exposure scenarios

The EFSA harmonized approach has also been used for the dietary intake assessment analysis [29]. Data on alcohol consumption for the groupings of beer, wine, spirits and unrecorded alcohol were obtained from the WHO GISAH [33] based on data from the FAO as well as other sources (e.g. country records) [34,35] for the year 2003 for populations older than 15. The data on unrecorded alcohol consumption were based on the estimated volume for the years after 1995 and populations older than 15 [36].

The distribution of beverage groups in the spirits category was taken from a national survey carried out in Brazil [37]. Consumption of tiquira was estimated based on data from the IBGE, which stated the annual tiquira consumption at 640,000 l [38].

The EC content of the alcoholic beverages was estimated based on data from our literature review (see above). Special exposure scenarios were developed for light, moderate and heavy drinkers of cachaça and tiquira with comparison to beer drinkers. For these calculations, we used the typical drink sizes from the WHO GENACIS study [39,40], of 50 ml for spirits (cachaça, tiquira) and 350 ml for beer, as well as data from our survey in Pernambuco (see above).

## Results

### Occurrence of ethyl carbamate in alcoholic beverages of the Brazilian market

The literature review identified 16 studies on the occurrence of EC in alcoholic beverages available in Brazil. Thirteen of the studies included data on cachaça [4,17-19,41-49], four studies researched tiquira [19,41,47,50], one was about grape wine including sparkling wines [51], and one on orange press-liquor spirit [52]. There were limited analytical results on other beverages on the Brazilian market (e.g. whisky, fruit spirit, or grappa) [19,41]. The studies are summarized in Table 1; we also include the new results from our analysis on EC in 33 cachaça brands from Pernambuco.

**Table 1:** Literature data on ethyl carbamate in recorded and unrecorded alcoholic beverages from the market and from experimental studies

				Ethyl carbamate [mg/l]						
**Beverage group/Study**	**Sample/Sampling characterization**	**Production origin**	**N**	**Mean**	**Median**	**90^th ^percentile**	**95^th ^percentile**	**99^th ^percentile**	**Maximum**	**Percentage above 0.15**

**1. Cachaça**										

Farah Nagato et al. [41]	Recorded, column/pot still, Brazilian market	São Paulo State, Brazil	13	0.33	0.24	0.68	0.95	1.14	1.20	69%

Boscolo [49]	Recorded, Brazilian market	Different states, Brazil	84	0.90	-^a^	-	-	-	5.5	87%

Andrade-Sobrinho et al. [19]	Recorded, column/pot still, Brazilian market	Different states, Brazil	126	0.77 (pot still: 0.63; column still: 0.93)	0.479	-	-	-	5.70	79%

Lelis [42]	Recorded, un-recorded column/ Pot still, Brazilian market.	Different states, Brazil	75	0.38 (pot still: 0.40; column still: 0.29)	0.36	0.63	0.83	0.91	0.95	87%

Baffa Júnior et al. [43]	Recorded, Brazilian market	Minas Gerais State, Brazil	22	1.20	0.60	1.67	1.96	10.19	12.38	77%

EFSA 2007 [4]	Recorded, European market	No data	19	0.229	0.11	-	0.478	-	0.73	-

Barcelos et al. [44]	Pot still, experimental	Minas Gerais State, Brazil	52	0.243	-	-	-	-	0.643	-

Bruno et al. [45]	Recorded, column/ pot still, experimental, Brazilian market	Rio de Janeiro State, Brazil	34	0.17 (pot still: 0.11; column still: 0.31)	0.10	0.42	0.60	0.68	0.71	44%

Labanca et al. [46]	Recorded, pot still Brazilian market,	Minas Gerais State, Brazil	69	0.89	0.79	1.78	2.10	2.42	2.61	93%

Andrade Sobrinho et al. [47]	Recorded, Sampling 2002	Sao Paulo State, Brazil	108	0.14	0.07	0.29	0.55	1.23	1.39	27%

Andrade Sobrinho et al. [47]	Recorded, sampling 2004	Different States, Brazil	36	-	0.108	-	-	-	0.46	33%

Andrade Sobrinho et al. [47]	Recorded, Brazilian market, sampling 2005	Brazil	41	-	0.163	-	-	-	1.16	58%

Andrade Sobrinho et al. [47]	Recorded, sampling 2006	Different States, Brazil	34	-	0.085	-	-	-	0.646	24%

Andrade Sobrinho et al. [47]	Recorded, Brazilian market, sampling 2006	Brazil	35	-	0.138	-	-	-	1.67	49%

Nóbrega et al. [17]	Recorded, pot still Brazilian market	Paraíba State, Brazil	25	0.22	0.20	0.40	0.42	0.63	0.70	68%

Lachenmeier et al. [18]	Recorded, un-recorded, column/ pot still, European/Brazilian markets	Different States, Brazil	42	0.27 (pot still: 0.15; column still: 0.37)	0.18	0.55	0.68	1.27	1.54	56%

Reche and Franco [48]^b^	Pot still, experimental	Brazil	73	0.11	0.05	0.24	0.29	0.83	1.29	15%

Reche and Franco [48]^b^	Column still, experimental	Brazil	42	0.42	0.41	1.79	2.10	2.93	3.37	83%

This study	Recorded, column/ pot still Brazilian market	Pernambuco State, Brazil	33	0.18 (pot still: 0.06; column still: 0.30)	0.19	0.33	0.39	0.49	0.53	55%

**2. Tiquira**										

Boscolo et al. [50]	No data	Maranhão State, Brazil	12	3.51	3.26	6.01	7.95	9.75	10.2	100%

Farah Nagato et al. [41]	No data	Maranhão State, Brazil	1	0.80	-	-	-	-	-	-

Andrade-Sobrinho et al. [19]	No data	Maranhão State, Brazil	37	2.35	1.80	5.36	6.10	8.60	10.00	100%

Andrade Sobrinho et al. [47]	No data	Maranhão State, Brazil	45	2.06	1.51	4.67	6.01	8.41	10.2	98%

**3. Other Beverages**										

Farah Nagato et al. [41]	Whisky/Fruit spirit, Brazilian market	Scotland/ Brazil.	2	0.7 (Scotch), 1.41 (Fruit spirit)	-	-	-	-	-	-

Andrade-Sobrinho et al. [19]	Grappa, Italian/ Brazilian markets.	Italy and Brazil	6	0.02	0.01	0.05	0.06	0.07	0.07	

Andrade-Sobrinho et al. [19]	Whisky, Brazilian market	USA and Scotland	19	0.14	0.10	0.22	0.35	0.39	0.40	26%

Francisquetti et al. [51]	Wines including sparkling wines, Brazilian market	R. Grande do Sul St, Brazil	124	0.004 to 0.019	-	-	-	-	0.07	0%

Ferreira et al. [52]	Orange press liquor spirit, experimental, not commercially available	Brazil	10	n.d.^c^	-	-	-	-	-	0%

^a ^Values marked as (-) not calculable because raw data is not available

^b ^Values were reported in g/hl of pure alcohol. Own recalculation to mg/l assuming an average alcoholic strength of 40% vol.

^c ^not detectable

For the purposes of our risk assessment, we summarized the single studies into an overall mean, median, and percentiles. The results of this meta analysis for the different classes of alcoholic beverages are presented in Table 2. According to the EFSA criteria [29], we provide scenarios for average content as well as the percentiles in all cases.

**Table 2:** Meta analysis on the ethyl carbamate occurrence in Brazilian spirits

			Ethyl carbamate [mg/l]				
**Type of beverage**	**Data sources**	**N**	**Mean**	**Median**	**90^th ^percentile**	**95^th ^percentile**	**99^th ^percentile**

Pot still cachaça	[17,18,42,45,46,48], this study	275	0.38	0.21	0.96	1.32	2.21

Column still cachaça	[18,42,45,48], this study	101	0.49	0.32	1.01	1.68	2.30

All types cachaça (without weighting for distillation type)	[17,18,41-43,45-48], this study	536	0.38	0.19	0.91	1.34	2.22

All types cachaça (with weighting according to production amount: 38% pot and 62% column still)	[17,18,45,46,48], this study	376	0.45	0.28	0.99	1.54	2.26

Regulated cachaça (hypothetical distribution after full implementation of 0.15 mg/l limit)^a ^	All types cachaça, see above	536	0.11	0.15	0.15	0.15	0.15

Tiquira	[19,41,47,50]	95	2.34	1.72	5.44	6.10	10.20

^a ^To derive this hypothetical distribution, all samples with concentrations above the limit were set to 0.15 mg/l

The meta analysis aimed to only include products available to the typical consumer, therefore we excluded the study on orange press liquor, which is currently not commercially available [52]. We also excluded studies that did not present the raw results but only averages [44,49,51]. Although the studies of Bruno et al. [45] and Reche and Franco [48] contain results of distillate fractions sampled in distilleries, we decided to include these studies with the aim of making the estimates more stable with the inclusion of more data.

For cachaça, it became quickly evident that it was problematic to simply average over all values, as the two major categories of cachaça - pot still and column still cachaça - appear to have significant differences in their EC contents. From the 13 studies on cachaça, three provided differentiated data on column still cachaça [18,45,48], and five provided data on pot still cachaça [17,18,45,46,48]. Our own results from Pernambuco also differentiated the two types. The rest of the studies gave no information on cachaça type and could not be used for meta analysis on type.

In total, we gathered 275 results on pot still cachaça (average 0.38 mg/l) and 101 results on column still cachaça (average 0.49 mg/l). Column still cachaça had a higher average EC content than pot still (t-test, p = 0.048). Including all analytical data from all selected studies, we had a total of 536 analytical results, with an average of 0.38 mg/l. However, this form of data summarization could lead to bias towards lower EC contents, as considerably more results on pot still than on column still cachaça were available. To avoid this bias, we also conducted a weighting according to production amount (Table 2). According to the Cachaça Official Guide [53], Brazilian production consists of 38% pot still (artisanal) and 62% column still (industrial) cachaça. We, therefore, used this percentage for weighting the total of 376 cachaças with known production methodology. The average content for the calculation with weighting was 0.45 mg/l.

To evaluate the potential effectiveness of the Brazilian 0.15 mg/l limit for cachaça, we also included a hypothetical calculation in Table 2, in which all samples with EC contents higher than the limit were set to 0.15 mg/l.

The meta analysis for tiquira resulted in 95 samples with an average content of 2.34 mg/l. For all other alcoholic beverages, the sample numbers were too low to derive an overall mean for the Brazilian market. There were no studies available for beer. It is, however, known from international studies, that beer and wine contain only very low concentrations of EC. Moreover, no significant geographical differences were found for beer and wine. We therefore decided to use the international average values (e.g. as published by EFSA [4]) to evaluate the missing groups in Brazil (Table 3).

**Table 3:** Ethyl carbamate occurrence in European-style alcoholic beverages from large international samplings (data from EFSA [4])

	Ethyl carbamate [mg/l]		
**Type of beverage**	**Mean**	**Median**	**95^th ^percentile**

Beer	0.005	0.005	0.006

Wine	0.007	0.005	0.078

Spirits (excluding fruit spirits)	0.094	0.022	0.390

- Whisky	0.040	0.030	0.106

- Rum	0.017	0.012	0.045

- Vodka	0.008	0.005	0.017

- Brandy	0.078	0.045	0.345

**Figure 1:**
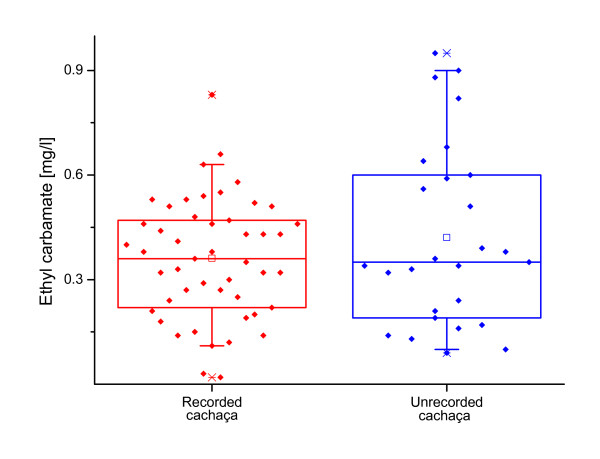
**Distribution of ethyl carbamate in recorded and unrecorded cachaça samples (data from Lelis **[33]**)**. No statistically significant differences between both collectives could be proven.

Finally, as significant unrecorded consumption occurs in Brazil, we also estimated the EC content in unrecorded alcoholic beverages. Considering the high likelihood that the majority of unrecorded consumption would be in the form of artisanally produced cachaça (i.e. pot still cachaça), we chose to use the contents of pot still cachaça for the category of unrecorded alcohol. The appropriateness of using the values of recorded pot still cachaça for unrecorded cachaça was also confirmed by two studies in the literature. Our own recent study included one sample of unrecorded cachaça, which had an EC content of 0.47 mg/l [18]. The study of Lelis [42] compared 27 unrecorded with 48 recorded cachaça samples. The average contents were 0.42 mg/l for unrecorded and 0.36 mg/l for recorded. Due to the relatively high standard deviations (Figure 1), no significant differences between both collectives could be proven (p = 0.236, own t-test calculation with data from Lelis [42]). The averages for these limited surveys of unrecorded cachaça therefore appeared to be in reasonable agreement with our meta analysis for pot still cachaça, and justified the use of this data for exposure estimation, until more comprehensive surveys on unrecorded alcohol are available.

### Exposure assessment

As the occurrence data in tables 2 and 3 show, the EC contents in beverages on the Brazilian market significantly depend on beverage type. The highest average contents were found in tiquira, by almost a factor of 5 or more than in cachaça. All other types of alcoholic beverages generally showed contents of less than 0.15 mg/l. For this reason, the population based exposure assessment separately considered the consumption of the different kinds of beverages. Table 4 shows our estimation on the annual consumption of the different categories, based on the WHO GISAH data [33] along with results from a national survey on spirit type consumed in the previous year [37]. The EC exposure due to alcoholic beverage consumption (Table 5) was calculated with the combined data from tables 2, 3, 4.

**Table 4:** Annual per capita consumption of alcoholic beverages in Brazil

Type of beverage	Annual per capita consumption [Litres of pure alcohol]^a^	Distribution in spirits category according to national survey^b^	Distribution in spirits category, own estimation^c^	Annual per capita consumption of spirits, own estimation [Litres of pure alcohol]^d^
Beer	3.41			

Wine	0.29			

Spirits all	2.06			

- Cachaça	(no data)	66%	66%	1.36

- Whisky	(no data)	24%	8.9%	0.18

- Rum	(no data)	13%	4.8%	0.10

- Vodka	(no data)	28%	10.3%	0.21

- Brandy	(no data)	23%	8.5%	0.17

- Other	(no data)	4%	1.5%	0.03

- Tiquira	(no data)	(no data)	(less than 1%)	0.003

Unrecorded	3.00			

^a ^Data from WHO GISAH for 2003 for population older than 15 [33]

^b ^Data from a national survey on types of spirits consumed in the previous year [37]. The total is not 100% because of overlapping.

^c ^Estimation was necessary to come to a total 100%. This was conducted on the basis of a 66% cachaça consumption. The rest of 34% was distributed according to the percentages from the national survey [37].

^d ^Calculated from the total spirits consumption of 2.06 l according to WHO GISAH [33] using the estimated distribution. The estimation for tiquira was based on annual consumption of 640.000 l from IBGE [38].

**Table 5:** Exposure with ethyl carbamate from alcoholic beverages in Brazil.

	Scenario 1 for cachaça all types without weighting			Scenario 2 for cachaça all types with weighting			Hypothetical scenario 3 for regulated cachaça (including regulated unrecorded cachaça)		
**EC exposure**	**Mean**	**Median**	**95^th ^percentile**	**Mean**	**Median**	**95^th ^percentile**	**Mean**	**Median**	**95^th ^percentile**

Beer	17.3	17.3	20.8	(similar to scenario 1)			(similar to scenario 1)		

Wine	0.77	0.55	8.61	(similar to scenario 1)			(similar to scenario 1)		

Cachaça	59.0	29.5	208.0	69.9	43.5	239.1	17.1	23.3	23.3

Whisky	0.82	0.62	2.18	(similar to scenario 1)			(similar to scenario 1)		

Rum	0.19	0.14	0.51	(similar to scenario 1)			(similar to scenario 1)		

Vodka	0.19	0.12	0.41	(similar to scenario 1)			(similar to scenario 1)		

Brandy	1.51	0.87	6.70	(similar to scenario 1)			(similar to scenario 1)		

Other	0.32	0.08	1.34	(similar to scenario 1)			(similar to scenario 1)		

Tiquira	0.80	0.59	2.09	(similar to scenario 1)			(similar to scenario 1)		

Unrecorded	130.1	71.9	452.1	(similar to scenario 1)			37.7	51.4	51.4

Total alcohol	211.1	121.7	702.7	221.9	135.7	733.7	76.7	94.9	117.3

Total plus other foods^a^	227.8	138.4	719.4	238.6	152.4	750.4	93.4	111.6	134.0

Calculated as ng/kg bw/day (calculated for a 60 kg person)

^a ^an exposure of 16.7 ng/kg bw/day is assumed for other foods by EFSA based on an international JECFA estimate, see [4,5] for details.

The highest exposures arose from cachaça (60 -70 ng/kg bw/day on average) and unrecorded alcohol (50-130 ng/kg bw/day on average). The total exposure from all alcoholic beverages was calculated to be around 100-200 ng/kg bw/day on average.

### Derivation of toxicological dose descriptors for ethyl carbamate

The literature search revealed two international risk assessments by the JECFA [5] and EFSA [4], as well as articles by O'Brien et al. [54] and Schlatter et al. [55] that contained data on toxicological dose descriptors for EC. The basis for all four evaluations was the 2004 NTP 2-year rodent bioassay [56] that contains data meeting the criteria for the modelling of the dose-response relationship for lifetime exposure to EC [5]. The BMDL value for EC obtained by the JECFA for the incidence of alveolar and bronchiolar neoplasms (the most sensitive endpoints) in male and female mice was found to be 0.3 mg/kg bw/day [5]. Similar results for dose-response modelling were published in the other studies [54,55]. The EFSA chose the same internationally established BMDL value of 0.3 mg/kg bw/day for their risk assessment of EC [4]. We also chose to use this internationally established value as the basis for our risk evaluation in Brazil.

Neither the JECFA nor EFSA published a T25 value for EC. However, O'Brien et al. [54] and Schlatter et al. [55] calculated the T25 dose descriptor from the same NTP data. The lowest dose group with a significantly increased tumour incidence was 1.2 mg/kg bw/day in male mice. At this dose, the cancer incidence increased by 30.2% compared to the control group. This lead to a T25 value of 1.0 mg/kg bw/day. The human dose descriptor HT25 could then be calculated from the T25 by dividing the animal dose data with the appropriate scaling factor. The scaling factor, calculated according to Sanner et al. [31] with data of the animal weights from NTP [56], was 6.3 leading to a HT25 of 0.16 mg/kg bw/day.

### Risk characterization

The exposure data from Table 5 was used to characterize risk using the MOE calculated from the BMDL (Table 6) and the lifetime cancer risk calculated with the T25 method (Table 7). As noted above, the threshold of 10,000, generally used for characterizing a public health risk, was used in our analysis (see discussion below).

**Table 6:** Margin of Exposure (MOE) for ethyl carbamate in different exposure scenarios.

	Scenario 1 for cachaça all types without weighting			Scenario 2 for cachaça all types with weighting			Hypothetical scenario 3 for regulated cachaça (including regulated unrecorded cachaça)		
**EC exposure**	**Mean**	**Median**	**95^th ^percentile**	**Mean**	**Median**	**95^th ^percentile**	**Mean**	**Median**	**95^th ^percentile**

Beer	17340	17340	14450	(similar to scenario 1)			(similar to scenario 1)		

Wine	388374	543724	34854	(similar to scenario 1)			(similar to scenario 1)		

Cachaça	5085	10170	1442	4294	6901	1255	17567	12882	12882

Whisky	365000	486667	137736	(similar to scenario 1)			(similar to scenario 1)		

Rum	1545882	2190000	584000	(similar to scenario 1)			(similar to scenario 1)		

Vodka	1564286	2502857	736134	(similar to scenario 1)			(similar to scenario 1)		

Brandy	198190	343529	44808	(similar to scenario 1)			(similar to scenario 1)		

Other	931915	3981818	224615	(similar to scenario 1)			(similar to scenario 1)		

Tiquira	374359	509302	143607	(similar to scenario 1)			(similar to scenario 1)		

Unrecorded	2305	4171	664	(similar to scenario 1)			7964	5840	5840

Total alcohol	1421	2466	427	1352	2212	409	3913	3161	2559

Total plus other foods	1317	2168	417	1257	1969	400	3213	2688	2240

Calculated with BMDL of 0.3 mg/kg bw/day (MOE = BMDL/Exposure).

**Table 7:** Lifetime cancer risk of ethyl carbamate in different whole population exposure scenarios.

	Scenario 1 for cachaça all types without weighting			Scenario 2 for cachaça all types with weighting			Hypothetical scenario 3 for regulated cachaça (including regulated unrecorded cachaça)		
**EC exposure**	**Mean**	**Median**	**95^th ^percentile**	**Mean**	**Median**	**95^th ^percentile**	**Mean**	**Median**	**95^th ^percentile**

Beer	2.7E-05	2.7E-05	3.2E-05	(similar to scenario 1)			(similar to scenario 1)		

Wine	1.2E-06	8.6E-07	1.3E-05	(similar to scenario 1)			(similar to scenario 1)		

Cachaça	9.2E-05	4.6E-05	3.3E-04	1.1E-04	6.8E-05	3.7E-04	2.7E-05	3.6E-05	3.6E-05

Whisky	1.3E-06	9.6E-07	3.4E-06	(similar to scenario 1)			(similar to scenario 1)		

Rum	3.0E-07	2.1E-07	8.0E-07	(similar to scenario 1)			(similar to scenario 1)		

Vodka	3.0E-07	1.9E-07	6.4E-07	(similar to scenario 1)			(similar to scenario 1)		

Brandy	2.4E-06	1.4E-06	1.0E-05	(similar to scenario 1)			(similar to scenario 1)		

Other	5.0E-07	1.2E-07	2.1E-06	(similar to scenario 1)			(similar to scenario 1)		

Tiquira	1.3E-06	9.2E-07	3.3E-06	(similar to scenario 1)			(similar to scenario 1)		

Unrecorded	2.0E-04	1.1E-04	7.1E-04	(similar to scenario 1)			5.9E-05	8.0E-05	8.0E-05

Total alcohol	3.3E-04	1.9E-04	1.1E-03	3.5E-04	2.1E-04	1.1E-03	1.2E-04	1.5E-04	1.8E-04

Total plus other foods	3.6E-04	2.2E-04	1.1E-03	3.7E-04	2.4E-04	1.2E-03	1.5E-04	1.7E-04	2.1E-04

Calculated with HT25 of of 0.16 mg/kg bw/day (Lifetime cancer risk = Exposure/HT25·0.25).

In the case of EC, the MOEs were below the 10,000 threshold for cachaça with the exception of the median exposure for scenario 1 (calculation without weighting). Notably, the values for the hypothetical scenario of regulated cachaça were above 10,000. For unrecorded alcohol, all scenarios were below 10,000. The cumulative exposures from all groups of alcoholic beverages and other foods resulted in levels below 10,000 with MOE values ranging between 400 and 2,466.

According to the T25 method, if average values for EC concentration in the beverages were assumed, the cancer risk was approximately 3 cases per population of 10,000. This risk went as high as 1 case per population of 1000, if alcoholic beverages with extremely high levels of EC were to be consumed on a daily basis (95th percentile). However, we think that this latter scenario, which is almost approaching the risk of ethanol in alcoholic beverages itself (in the extreme case up to 1:100 for females drinking more than 100 g on average for all alcohol-attributable cancers combined based on a different approach [57]), overestimates the risk of EC, as it is relatively unlikely that highly contaminated alcohol is consumed on a daily basis. In light of the epidemiological evidence regarding alcoholic beverage consumption, we think that an average risk in the range of 10^-4 ^is probable. The estimation of a risk in the range of 10^-4 ^is based on indirect reasoning. For if the risk was higher it would be expected to result in markedly elevated cancer rates for regions with high EC exposure (e.g. the tiquira-consuming states of Maranhão and Piauí, Northeast Brazil) compared to regions with the same per capita consumption but lower EC exposure, which until now has not been the case.

**Figure 2:**
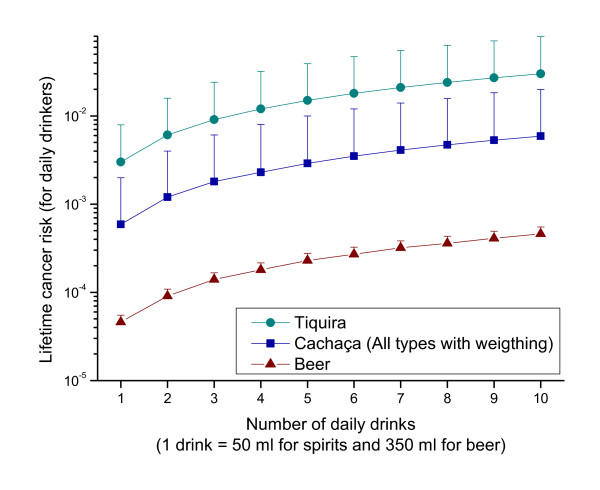
**Lifetime cancer risk calculated with the T25 method for consumers of different alcoholic beverages in Brazil (Mean with 95th percentile as error bar are shown)**.

In addition to this whole population estimate, we provide individual risk assessments for daily drinkers of cachaça, tiquira and beer (Figure 2). For one drink per day, the average EC exposure would be 0.4 μg/kg bw/day (cachaça), 1.95 μg/kg bw/day (tiquira) or 0.03 μg/kg bw/day (beer). The MOE for these exposures are 800 (cachaça), 154 (tiquira) and 10,286 (beer). The corresponding lifetime cancer risks would be 5.9E-04 (cachaça), 3.0E-03 (tiquira) and 4.6E-05 (beer).

**Figure 3:**
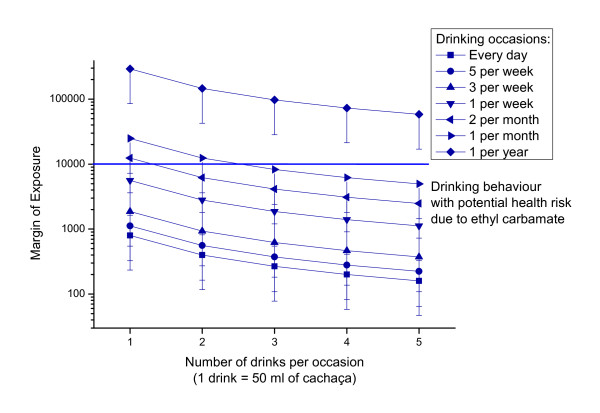
**Margin of Exposure calculated using the BMD-method for different exposure scenarios of cachaça consumption in Brazil (calculated for all types cachaça with weighting, mean with 95th percentile as error bar are shown)**.

For cachaça, we also provide a more detailed evaluation showing the MOE resulting from different drinking scenarios (once a year to once daily) as well as for different numbers for drinks per occasion (Figure 3). MOEs below 10,000 were calculated for cachaça consumers drinking more than 1 drink per week or more than 2 drinks on two occasions per month.

## Discussion

### Limitations of the risk assessment

The first and major limitation of this risk assessment is the extrapolation step from animal to human data. In this, it may be especially scrutinized that the incidence of alveolar and bronchiolar adenoma or carcinoma were used as critical responses for obtaining the toxicological dose descriptors [5]. These sites are not considered to be alcohol-associated in human epidemiology [1]. However, the JECFA noted that the ranges of BMDL values of other cancer sites are in the same range as those of the alveolar and bronchiolar adenoma or carcinoma [5], and we agree that for precautionary reasons the conservative lower end of the range of values should be used. As a multi-site carcinogen, it is interesting and relevant that EC was proven in rodents and non-human primates to induce tumours in the liver, which is also a proven site of carcinogenicity of alcoholic beverages in general [1]. EC also induces squamous cell papilloma or carcinoma in the forestomach of mice, which contain comparable tissues to the mouth and oesophagus of humans, which are also known sites for the carcinogenicity of alcoholic beverages in general, likely impacted by the ethanol-acetaldehyde pathway [1]. Animal experiments point to complex interactions between ethanol and EC, e.g. in decreased first-pass hepatic clearance [12]. However, no consistent trend of the co-administration of ethanol on the carcinogenicity of EC has been found [5]. Nevertheless, there remains the possibility that the effects of both agents are additive. We also cannot exclude the possibility of synergistic effects as seen with the combined risk of alcohol and tobacco consumption [1,58,59]. Interactions with acetaldehyde, a further carcinogen directly contained in alcoholic beverages and formed during ethanol metabolism must also be considered [60]. However for our quantitative risk assessment, the current state of knowledge did not allow for the consideration of the interactions of EC with ethanol and other substances.

The second limitation concerns the occurrence data of EC in foods and beverages in Brazil. Despite the considerable knowledge on cachaça, there were few studies on other beverages and no studies on other fermented foods. Therefore, our risk assessment contains data extrapolated from international surveys conducted in other countries. We therefore cannot exclude the possibility that other beverages or foods might contain higher concentrations of EC, in which case we may have underestimated the risk. In the current evaluation, the major contributing factors to EC exposure were cachaça and unrecorded alcohol, while the other exposures could be quantitatively neglected. Therefore, we would currently exclude the possibility of having overestimated the risk.

For cachaça itself, we have the problem that the market is so large, that even the 500 samples we have considered in our meta analysis may appear to be quite small a number. However, during our calculations it became evident that the inclusion or exclusion of single studies led to only minor changes in the overall averages (only on the second decimal place), as such we think we have reached a very stable estimate of the current situation. This also appears to justify the inclusion of studies sampling distillates and not products bottled and sold to the final consumer. A larger problem was the differences between the different categories of cachaça, which we think we have adequately addressed by using different calculation approaches (e.g. with and without weighting of the distillation type). In total, the differences between the averages of the calculation methods remained relatively small (i.e. 0.1 mg/l). In the population based risk assessments, the differences that arose in the MOE values (e.g. MOE 5058 without weighting, 4294 with weighting) were not relevant for the interpretation, as both values were significantly below the threshold of 10,000 (see below).

All factors considered, the mentioned limitations were not as grave as to completely prohibit a risk assessment of EC. It is in fact on the contrary, as the same limitations generally apply to the assessments of the international agencies EFSA and JECFA, whose approaches we specifically followed.

### Risk of ethyl carbamate for the Brazilian population

Our risk assessment comes to the conclusion that EC may pose a health risk for the alcohol-drinking population in Brazil. MOEs can be used by risk managers for setting priority, with a small MOE representing a higher risk than a larger one. In general, a MOE of 10,000 or higher, if based on a BMDL from an animal study, would be considered a low public health concern and subsequently a low priority for risk management actions [29]. The MOEs for cachaça or unrecorded alcohol alone are below 10,000. If we look at the cumulative exposure from all sources, this leads to MOEs around 1,300 and lifetime cancer risks in the 1:10,000 range. These are considerably higher risks than what the EFSA has calculated for Europe, where the MOE for the overall population would be in the range of 9,090 to 5,450. Even these values for Europe were considered as health relevant by EFSA. The difference between Europe and Brazil derives from the fact that the preferred alcoholic beverages in Europe have relatively low contents of EC, while in Brazil the preferred alcoholic beverages with the exception of beer contain comparably high concentrations of EC.

### Risk for individual drinkers

On an individual scale, the highest risks arose for regular drinkers of EC contaminated beverages (e.g. compare cachaça and beer in Figure 2). The health relevant range was reached even for moderate drinkers of 1 drink of cachaça per day (MOE 800), as well as for binge drinkers that drink more than three drinks on one occasion per month (MOE 8267). From our survey in Pernambuco, the binge drinkers that consumed quartinhos on a daily basis appeared to be at highest risk, e.g. one quartinho (170 ml) per day would correspond to a MOE of 235, and the maximum amount observed of four quartinhos (680 ml) would lead to a MOE of 59. In terms of drinking patterns and prevalence, it should be noted that a survey carried out in Bahia's capital (Salvador) showed the prevalence of high-risk drinking at 6.9% [25]. A recent national survey found that of the total sample (including those who were abstinent) 28% reported at least one occasion of binge drinking in the 12 months prior to the interview [27].

Similar to the results on a population scale, the risks for individual drinkers in Brazil appeared to be higher than the ones in Europe from the EFSA study, which stated MOEs of 4620 to 8110 for consumers of alcoholic beverages in general, and specifically MOEs of 3000 to 17,600 for consumers of beer (1000 ml daily), and a MOE of 5000 for consumers of spirits (125 ml daily).

An interesting finding of this study is the fact that tiquira consumers were at an even higher risk than consumers of cachaça. Tiquira appears to be the spirit with the highest EC contamination worldwide. Even stone fruit spirits that were thus far considered as the beverage group highest in EC, usually contain less than 1 mg/l of EC and only very seldom more than 1.5 mg/l [11]. The EFSA calculated a MOE of 540 for individual consumers of fruit spirits at the 95^th ^percentile (i.e. 125 ml daily). Our corresponding calculation for individual consumers of tiquira lead to a MOE of 154 for one drink per day and up to a MOE of 15 for ten drinks per day. Therefore, the MOE has almost reached unity meaning that the human exposure (which is ranging between 2 to 20 μg/kg bw/day) has almost reached the dose that may cause a 10% cancer incidence in experimental animals.

In evaluating these risks, we should again mention and take into consideration that EC is not the only compound from alcoholic beverages, which may cause cancer in humans [1]. The IARC has concluded that consumption of alcoholic beverages is carcinogenic to humans (see introduction) [1]. Based on these assessments, it was estimated that alcohol caused almost 500,000 cancer deaths worldwide in 2004 [61]. The main pathway is probably acetaldehyde [60] and as has been discussed EC may also play a significant role.

### Policy implications

In light of the proven public health risk of EC in cachaça signified by MOE values below 10,000, it is commendable that Brazil has implemented a legislative limit for cachaça. Table 6 shows that the full implementation of this limit would lead to MOE values above 10,000 for cachaça alone. Therefore, the policy appears to be toxicologically founded and effective if it can be implemented and enforced. Although there is no information about the association between liver cancer and cachaça available, a case-control study carried out in Brazilian cities showed that consumption of cachaça had a high risk for cancers of the upper aerodigestive tract [62]. It is with great likelihood that EC contributed to this risk.

A major problem here is the large consumption of unrecorded alcohol (which we assume to be in the form of illegally-produced and artisanally-produced pot-still cachaça). This consumption is so high that even assuming unrecorded producers also apply measures to bring the EC content to the limit of 0.15 mg/l, it would still not effectively bring the MOE of unrecorded alcohol above 10,000. Moreover, in the cumulative assessment of all alcoholic beverages and other foods, the full implementation of the limit for both cachaça and unrecorded alcohol also does not appear to be sufficient for bringing the exposure to a level outside of the health-relevant range of 10,000. However, it would at least increase the MOE by a factor of 3-6 and therefore provide a quantifiable increased safety measure for the Brazilian population. We therefore recommend that the limit should be enacted as planned in June 2010, and that opposing opinions from the alcohol industry be overruled due to significant and demonstrable public health risk.

The problem of EC contamination of unrecorded alcohol also strengthens the point that alcohol policy not neglect this type of consumption [63]. The Brazilian Beverage Association estimated that approximately 50% of all spirits consumed in Brazil were unrecorded in 1984 [64]. According to a more recent WHO estimation, 59% of spirits and 34% of total alcohol consumption is unrecorded (see table 4). Here we have the additional danger that cachaça producers unable to meet the EC limit simply move to the unrecorded market, a situation which is already compounded by the relatively high taxation of spirits in Brazil compared to other middle income countries [65]. In some areas of Brazil, it has been estimated that only about 10% of all distilleries are registered [66]. Indeed, there is scant information regarding unrecorded alcohol consumption and this issue demands further investigation [3].

It must also be appreciated that from a quantitative standpoint the real risk factor of alcoholic beverages is of course not EC, but ethanol or its metabolite acetaldehyde, which when combined have a risk of at least one order of magnitude higher (i.e. 10^-3^-10^-2^) [57]. For comparison, the MOE for ethanol in alcoholic beverages was judged to be 3 (for an exposure of 22.8 ml ethanol) [67]. Naturally, scientifically proven effective measures for reducing alcohol consumption itself [68,69] would also simultaneously lead to a reduced EC exposure. We think that both measures - mitigation of the EC problem as well as alcohol policy measures - should go hand in hand.

Regarding policy, we also advise the implementation of mitigative measures and possibly a limit for tiquira, as this beverage may be problematic for a sub-group of the population.

## Conclusions

The case of EC contamination in alcoholic beverages from Brazil, particularly in cachaça and tiquira, is extremely relevant, as we have found a considerably higher exposure than in studies conducted in Europe [4] or Central America [70]. In our opinion the scientific evidence is sufficient grounds for the strict enforcement of the maximum limit for EC in cachaça, and the implementation of such an effort for tiquira. Priority research should also be conducted for gaining better knowledge on the formation mechanisms and strategies for EC reduction in these beverages as well as for investigating other popular Brazilian alcoholic beverages (e.g. Brazilian vodkas and rums) that may also contain EC. Furthermore, measures that are applicable in small-scale artisanal distilleries should be developed and distilleries (including the unrecorded producers) should be made aware about the risk and possibly receive mitigative measures.

From an epidemiological point of view, it may be sensible to study cancer incidences of ethanol, acetaldehyde and EC related cancer sites prior to the implementation of the cachaça limit and in the following years. This would also provide an excellent case for studying the effectiveness of food policy measures regarding carcinogenic contaminants as well as the validity of risk evaluations using the MOE approach.

However, first and foremost, the safety of consumers should be improved by taking the appropriate means to reduce known risk factors such as EC from beverages. The current knowledge clearly is sufficient to necessitate intervention, given the precautionary principle of public health.

## List of abbreviations

BMD: benchmark dose for a benchmark response of 10%; BMDL/BMDL10: Benchmark Dose Lower Confidence Limit for a benchmark response of 10% (The BMDL is an estimate of the lowest dose that is 95% certain to cause no more than a 10% effect); EC: ethyl carbamate; EFSA: European Food and Safety Authority; FAO: Food and Agriculture Organization; GISAH: Global Information System on Alcohol and Health; HT25: human tumorigenic dose 25%, which is converted from the T25 by dividing it with the appropriate scaling factor for interspecies dose scaling based on comparative metabolic rates; IARC: International Agency for Research on Cancer; IBGE: Instituto Brasileiro de Geografia e Estatistica; JECFA: Joint FAO/WHO Expert Committee on Food Additives; MOE: margin of exposure; NTP: National Toxicology Program; T25: tumorigenic dose 25% (chronic dose of a carcinogen that leads in experimental animals to a tumour incidence of 25%); VSD: virtually safe dose; WHO: World Health Organization.

## Competing interests

The authors declare that they have no competing interests.

## Authors' contributions

DWL developed conception and design of the study, drafted the first version of the manuscript, organized data acquisition, and performed the meta analysis, MOE and T25 calculations. MCPL and FKC collected the alcohol consumption and exposure data for Brazil. ICCN conducted the literature review on EC in cachaça and provided EC occurrence and exposure data. ICCN and JAPP conducted analyses and provided original data on occurrence of EC in cachaça samples from Pernambuco State. MCPL, FKC and ICCN contributed to the interpretation of findings with their local knowledge about the specific situation in Brazil and revised the manuscript accordingly. FK participated in drafting and revising the manuscript and provided language editing. JR conceived of the study, provided the original contact between the members of the international research team, participated in the design and coordination of the study and helped to draft the manuscript. All authors read and approved the final manuscript.

## Pre-publication history

The pre-publication history for this paper can be accessed here:

http://www.biomedcentral.com/1471-2407/10/266/prepub

## References

[B1] Baan R Straif K Grosse Y Secretan B El Ghissassi F Bouvard V Altieri A Cogliano V WHO International Agency for Research on Cancer Monograph Working Group Carcinogenicity of alcoholic beverages Lancet Oncol 2007 8 292 293 10.1016/S1470-2045(07)70099-2 17431955

[B2] Secretan B Straif K Baan R Grosse Y El Ghissassi F Bouvard V Benbrahim-Tallaa L Guha N Freeman C Galichet L Cogliano V A review of human carcinogens - Part E: tobacco, areca nut, alcohol, coal smoke, and salted fish Lancet Oncol 2009 10 1033 1034 10.1016/S1470-2045(09)70326-2 19891056

[B3] Rehm J Kanteres F Lachenmeier DW Unrecorded consumption, quality of alcohol and health consequences Drug and Alcohol Review 2010 in press 10.1111/j.1465-3362.2009.00140.x 20636660

[B4] EFSA Ethyl carbamate and hydrocyanic acid in food and beverages EFSA J 2007 551 1 44

[B5] Vavasour E Renwick AG Engeli B Barlow S Castle L DiNovi M Slob W Schlatter J Bolger M Ethyl carbamate WHO Food Additives Series 55. Safety evaluation of certain contaminants in food. Prepared by the sixty-fourth meeting of the Joint FAO/WHO Expert Committee on Food Additives (JECFA) 2006 Geneva, Switzerland: WHO and FAO 205 316

[B6] Dennis MJ Howarth N Key PE Pointer M Massey RC Investigation of ethyl carbamate levels in some fermented foods and alcoholic beverages Food Addit Contam 1989 6 383 389 2721787 10.1080/02652038909373794

[B7] Battaglia R Conacher HBS Page BD Ethyl carbamate (urethane) in alcoholic beverages and foods: a review Food Addit Contam 1990 7 477 496 2203651 10.1080/02652039009373910

[B8] Schlatter J Lutz WK The carcinogenic potential of ethyl carbamate (urethane): risk assessment at human dietary exposure levels Food Chem Toxicol 1990 28 205 211 10.1016/0278-6915(90)90008-B 2188890

[B9] Zimmerli B Schlatter J Ethyl carbamate: analytical methodology, occurrence, formation, biological activity and risk assessment Mutat Res 1991 259 325 350 10.1016/0165-1218(91)90126-7 2017216

[B10] Sen NP Seaman SW Boyle M Weber D Methyl carbamate and ethyl carbamate in alcoholic beverages and other fermented foods Food Chem 1993 48 359 366 10.1016/0308-8146(93)90318-A

[B11] Lachenmeier DW Schehl B Kuballa T Frank W Senn T Retrospective trends and current status of ethyl carbamate in German stone-fruit spirits Food Addit Contam 2005 22 397 405 10.1080/02652030500073360 16019810

[B12] Beland FA Benson RW Mellick PW Kovatch RM Roberts DW Fang JL Doerge DR Effect of ethanol on the tumorigenicity of urethane (ethyl carbamate) in B6C3F1 mice Food Chem Toxicol 2005 43 1 19 10.1016/j.fct.2004.07.018 15582191

[B13] IARC IARC Monographs on the Evaluation of Carcinogenic Risks to Humans, Vol. 96, Alcoholic Beverage Consumption and Ethyl Carbamate (Urethane) Lyon, France in press PMC4781168 21735939

[B14] Cadranel JF Legendre C Desaint B Delamarre N Florent C Levy VG Liver disease from surreptitious administration of urethane J Clin Gastroenterol 1993 17 52 56 10.1097/00004836-199307000-00015 8409301

[B15] Conacher HBS Page BD Ethyl carbamate in alcoholic beverages: a canadian case history Proceedings of Euro Food Tox II 1986 Schwerzenbach, Switzerland: European Society of Toxicology 237 242

[B16] DOU Ministério da agricultura, pecuária e abastecimento, instrução normativa N° 13 de 29 de junho de 2005 Diário Oficial da União 2005 124 3 4

[B17] Nóbrega ICC Pereira JAP Paiva JE Lachenmeier DW Ethyl carbamate in pot still cachaças (Brazilian sugar cane spirits): Influence of distillation and storage conditions Food Chem 2009 117 693 697 10.1016/j.foodchem.2009.04.067

[B18] Lachenmeier DW Kuballa T Lima MCP Nóbrega ICC Kerr-Corrêa F Kanteres F Rehm J Ethyl carbamate analysis in German fruit spirits and Brazilian sugarcane spirits (cachaça): Improved sample cleanup with automated parallel evaporation Deut Lebensm Rundsch 2009 105 507 512

[B19] de Andrade-Sobrinho LG Boscolo M Lima-Neto BD Franco DW Ethyl carbamate in alcoholic beverages (cachaca, tiquira, whisky and grape) Quim Nova 2002 25 1074 1077

[B20] Venturini Filho WG do Prado Mendes B Fermentação alcoólica de raízes tropicais CULTURAS DE TUBEROSAS AMILÁCEAS LATINO AMERICANAS. Tecnologia, usos e potencialidades de tuberosas amiláceas Latino Americanas 2003 3 Paranavaí, PR, Brazil: Associação Brasileira dos Produtores de Amido de Mandioca 530 575

[B21] Furtado JLB Bezerra CWB Marques EP Marques ALB Cyanide in "tiquira": risks and analytical methodology Cienc Tecnol Aliment 2007 27 694 700

[B22] Polastro LR Boso LM Andrade-Sobrinho LG Lima-Neto BS Franco DW Nitrogen compounds in distilled beverages: cachaça and tiquira Cienc Tecnol Aliment 2001 21 78 81

[B23] Santos GS Marques EP Silva HAS Bezerra CWB Marques AB Identification and characterization of crystal violet in cassava spirits (tiquira) Quim Nova 2005 28 583 586

[B24] IBRAC - Brazilian Institute of Cachaça Mercado interno [domestic market] Brasília http://www.ibrac.net/index.php?view=article&catid=41%3Amercado-interno&id=46%3Amercado-interno&format=pdf&option=com_content&Itemid=47 Accessed: 2009-08-25

[B25] Almeida-Filho N Lessa I Magalhães L Araújo MJ Aquino E Kawachi I James SA Alcohol drinking patterns by gender, ethnicity, and social class in Bahia, Brazil Rev Saude Publica 2004 38 45 54 10.1590/S0034-89102004000100007 14963541

[B26] Coutinho EP Aspectos da evolução do mercado da cachaça [in Portuguese] Encontro Nacional de Engenharia de Produção 2003 Ouro Preto, MG, Brazil: ABEPRO 1 8

[B27] Laranjeira R Pinsky I Sanches M Zaleski M Caetano R Alcohol use patterns among Brazilian adults Rev Bras Psiquiatr 2009 in press 10.1590/s1516-44462009005000012 19918673

[B28] Abramson CI Howard W Zolna M Nain S Aquino IS Rocha H Moraes Z Page MM A price survey comparison of alcoholic beverages with the five basic food groups in Paraiba, Brazil J Soc Sci 2006 2 100 103 10.3844/jssp.2006.100.103

[B29] EFSA Opinion of the Scientific Committee on a request from EFSA related to a harmonised approach for risk assessment of substances which are both genotoxic and carcinogenic EFSA J 2005 282 1 31

[B30] Kodell RL Replace the NOAEL and LOAEL with the BMDL01 and BMDL10 Environ Ecol Stat 2009 16 3 12 10.1007/s10651-007-0075-3

[B31] Sanner T Dybing E Willems MI Kroese ED A simple method for quantitative risk assessment of non-threshold carcinogens based on the dose descriptor T25 Pharmacol Toxicol 2001 88 331 341 10.1034/j.1600-0773.2001.d01-126.x 11453374

[B32] Dybing E Sanner T Roelfzema H Kroese D Tennant RW T25: A simplified carcinogenic potency index: Description of the system and study of correlations between carcinogenic potency and species/site specificity and mutagenicity Pharmacol Toxicol 1997 80 272 279 10.1111/j.1600-0773.1997.tb01973.x 9225363

[B33] WHO Global Information System on Alcohol and Health 2009 Geneva, Switzerland. World Health Organization http://www.who.int/globalatlas/default.asp

[B34] Rehm J Rehn N Room R Monteiro M Gmel G Jernigan D Frick U The global distribution of average volume of alcohol consumption and patterns of drinking Eur Addict Res 2003 9 147 156 10.1159/000072221 12970583

[B35] Rehm J Klotsche J Patra J Comparative quantification of alcohol exposure as risk factor for global burden of disease Int J Methods Psychiatr Res 2007 16 66 76 10.1002/mpr.204 17623386 PMC6878477

[B36] Rehm J Room R Monteiro M Gmel G Graham K Rehn N Sempos CT Frick U Jernigan D Ezzati M, Lopez AD, Rodgers A, Murray CJL Alcohol use Comparative Quantification of Health Risks. Global and Regional Burden of Disease Attributable to Selected Major Risk Factors 2004 1 Geneva: World Health Organization 959 1108

[B37] Laranjeira R Pinsky I Zaleski M Caetano RI Levantamento nacional sobre os padrões de consumo de álcool na população brasileira [in Portuguese] 2007 Brasília, Brazil: Secretaria Nacional Antidrogas 20945019

[B38] IBGE Censo agropecuário 1995-1996 1998 Maranhão, Brazil: Instituto Brasileiro de Geografia e Estatística

[B39] Lima MCP Kerr-Corrêa F Tucci AM Simao MO Oliveira JB Cavariani MB Fantazia MM Gender differences in heavy alcohol use: A general population survey (the GENACIS project) of São Paulo City, Brazil Contemp Drug Probl 2007 34 427 444

[B40] Taylor B Rehm J Aburto JTC Bejarano J Cayetano C Kerr-Corrêa F Ferrand MP Gmel G Graham K Greenfield TK Alcohol, Gender, Culture and Harms in the Americas: PAHO Multicentric Study Final Report 2007 Washington, DC: Pan American Health Organization

[B41] Farah Nagato LA Silva OA Yonamine M Penteado MdVC Quantitation of ethyl carbamate (EC) by gas chromatography and mass spectrometric detection in distilled spirits Alimentaria 2000 31 31 36

[B42] Lelis VG Ocorrência de carbamato de etila e sua formação em cachaça de alambique e em aguardente de cana-de-açúcar 2006 Viçosa, Minas Gerais, Brazil: Universidade Federal de Viçosa

[B43] Júnior JCB Soares NFF Pereira JMdATK Melo NRd Ocorrência de carbamato de etila em cachaças comerciais da região da zona da Mata Mineira - MG Alim Nutr Araraquara 2007 18 371 373

[B44] Barcelos LVF das Graças Cardoso M Vilela FJ dos Anjos JP Teores de carbamato de etila e outros componentes secundários em diferentes cachaças produzidas em trés regiões do estado de Minas Gerais: Zona da mata, sul de minas e vale do jequitinhonha Quim Nova 2007 30 1009 1011

[B45] Bruno SNF Vaitsman DS Kunigami CN Brasil MG Influence of the distillation processes from Rio de Janeiro in the ethyl carbamate formation in Brazilian sugar cane spirits Food Chem 2007 104 1345 1352 10.1016/j.foodchem.2007.01.048

[B46] Labanca RA Gloria MBA Afonso RJDF Determination of Ethyl Carbamate in Sugar Cane Spirits by GC-MS Quim Nova 2008 31 1860 1864

[B47] de Andrade Sobrinho LG Cappelini LTD da Silva AA Galinaro CA Buchviser SF Cardoso DR Franco DW Teores de carbamato de etila em aguardentes de cana e mandioca. Parte II Quim Nova 2009 32 116 119

[B48] Reche RV Franco DW Distinction between cachaças distilled in pot stills and in columns using chemometrics Quim Nova 2009 32 332 336

[B49] Boscolo M Caramelo e carbamato de etila em aguardente de cana. Quantifição e ocorrência 2001 São Carlos, Brazil: Instituto de Química de São Carlos

[B50] Boscolo M Andrade-Sobrinho LG Lima-Neto BS Marques EP Franco DW Presença de carbamato de etila em aguardente de mandioca (tiquira) Engarrafador Moderno 1998 58 62 64

[B51] Francisquetti EL Vanderlinde R Carrau JL Moyna P Ethyl carbamate content in wines produced and commercialized in southern Brazil Acta Farm Bonaerense 2002 21 201 204

[B52] Ferreira JO Junior HR Faria JB The production of orange press liquor spirit: Technical and economic aspects Alim Nutr Araraquara 2008 17 1 6

[B53] Cachaça Official Guide (2005) - Annual Brazil 2005 Sabará, MG, Brazil: Folha de Sabará Empresa Jornalística Ltda

[B54] O'Brien J Renwick AG Constable A Dybing E Müller DJ Schlatter J Slob W Tueting W van Benthem J Williams GM Wolfreys A Approaches to the risk assessment of genotoxic carcinogens in food: a critical appraisal Food Chem Toxicol 2006 44 1613 1635 10.1016/j.fct.2006.07.004 16887251

[B55] Schlatter J DiNovi M Setzer RW Application of the margin of exposure (MoE) approach to substances in food that are genotoxic and carcinogenic: example: ethyl carbamate (CAS 51-79-6) Food Chem Toxicol 2010 48 Suppl 1 S63 S68 20113856 10.1016/j.fct.2009.10.032

[B56] NTP technical report on the toxicology and carcinogensis. Studies of urethane, ethanol, and urethane/ethanol (urethane, CAS No. 51-79-6; ethanol, CAS No. 64-17-5) in B6C3F1 mice (drinking water studies) Natl Toxicol Program Tech Rep Ser 2004 510 1 346 15625555

[B57] Rehm J Room R Taylor B Method for moderation: measuring lifetime risk of alcohol-attributable mortality as a basis for drinking guidelines Int J Methods Psychiatr Res 2008 17 141 151 10.1002/mpr.259 18763694 PMC6878565

[B58] Pelucchi C Gallus S Garavello W Bosetti C La Vecchia C Cancer risk associated with alcohol and tobacco use: focus on upper aero-digestive tract and liver Alcohol Res Health 2006 29 193 198 17373408 PMC6527045

[B59] Taylor B Rehm J When risk factors combine: the interaction between alcohol and smoking for aerodigestive cancer, coronary heart disease, and traffic and fire injury Addict Behav 2006 31 1522 1535 10.1016/j.addbeh.2005.11.008 16443330

[B60] Lachenmeier DW Kanteres F Rehm J Carcinogenicity of acetaldehyde in alcoholic beverages: risk assessment outside ethanol metabolism Addiction 2009 104 533 550 10.1111/j.1360-0443.2009.02516.x 19335652

[B61] Rehm J Mathers C Popova S Thavorncharoensap M Teerawattananon Y Patra J Global burden of disease and injury and economic cost attributable to alcohol use and alcohol-use disorders The Lancet 2009 373 2223 2233 10.1016/S0140-6736(09)60746-7 19560604

[B62] Schlecht NF Pintos J Kowalski LP Franco EL Effect of type of alcoholic beverage on the risks of upper aerodigestive tract cancers in Brazil Cancer Causes Control 2001 12 579 587 10.1023/A:1011226520220 11552705

[B63] Lachenmeier DW Reducing harm from alcohol: what about unrecorded products? Lancet 2009 374 977 10.1016/S0140-6736(09)61661-5 19766878

[B64] Galduróz JCF Caetano R Epidemiology of alcohol use in Brazil Rev Bras Psiquiatr 2004 26 Suppl 1 S3 S6 15729435 10.1590/s1516-44462004000500002

[B65] WHO Global Status Report: Alcohol Policy 2004 Geneva, Switzerland: World Health Organization

[B66] Vaissman M Haworth A, Simpson R Licit and illicit beverages in Brazil Moonshine Markets. Issues in unrecorded alcohol beverage production and consumption 2004 New York: Brunner-Routledge 87 102

[B67] Gold LS Ames BN Slone TH How Many Fold Lower Is Human Exposure Than the Dose That Gave Rodents Cancer: Margin of Exposure, MOE (Rodent Cancer Dose/Human Exposure). Berkeley, CA http://potency.berkeley.edu/MOEtable.html Accessed: 2009-08-11

[B68] Babor T Caetano R Casswell S Edwards G Giesbrecht N Graham K Grube J Hill L Holder H Homel R Alcohol: No ordinary commodity. Research and public policy 2010 2 Oxford: Oxford University Press

[B69] Rehm J Greenfield TK Public alcohol policy: current directions and new opportunities Clin Pharmacol Ther 2008 83 640 643 10.1038/sj.clpt.6100502 18305457 PMC2782936

[B70] Lachenmeier DW Kanteres F Kuballa T López MG Rehm J Ethyl carbamate in alcoholic beverages from Mexico (tequila, mezcal, bacanora, sotol) and Guatemala (cuxa): Market survey and risk assessment Int J Environ Res Public Health 2009 6 349 360 10.3390/ijerph6010349 19440288 PMC2672322

